# Supplementary material for: Thermal transport properties of porous silicon filled by ionic liquid nanocomposite system
Source: Sci Rep. 2023 Apr 11;13:5889. doi: 10.1038/s41598-023-32834-8 (PMC10090056; doi:10.1038/s41598-023-32834-8)
Supplement: Supplementary file 1 — Supplementary Information. [file 41598_2023_32834_MOESM1_ESM.docx]

Supporting Information

Thermal transport properties of porous silicon filled by ionic liquid nanocomposite system

Pavlo Lishchuk, Alina Vashchuk, Sergiy Rogalsky, Lesia Chepela, Mykola Borovyi,

David Lacroix, and Mykola Isaiev

**^1^H NMR analysis data for synthesized ionic liquids**

Triethylammonium bis(trifluoromethylsulfonyl)imide [TEA]-[TFSI]

^1^H NMR (400 MHz, CDCl_3_): δ 6.87 (s, 1H, NH), 3.17 (q, 6H, CH_2_), 1.31 (t, 9H, CH_3_)

Fig. S1. ^1^H NMR spectrum of [TEA] [TFSI]

Imidazolium bis(2-ethylhexyl)phosphate [Im] [BEHP]

^1^H NMR (400 MHz, CDCl_3_) δ 8.04 (s, 1H, C_2_-H), 7.12 (d, *J* = 1.2 Hz, 2H, C_4_-H, C_5_-H)), 3.8 (q, *J* = 4.9 Hz, 4H, OCH_2_), 1.54 (h, *J* = 5.9 Hz, 2H, CH_2_*CH*), 1.47 – 1.19 (m, 16H, CH_2_), 0.99 – 0.70 (m, 12H, CH_3_).

Fig. S2. ^1^H NMR spectrum of [Im] [BEHP]

1-butyl-3-methylimidazolium bis(trifluoromethylsulfonyl)imide [BMIm] [TFSI]

^1^HNMR (400 MHz, CDCl_3_) δ 8.72 (s, 1H, C_2_-H), 7.32 – 7.29 (m, 2H, C_4_-H, C_5_-H), 4.16 (t, J = 7.4 Hz, 2H, NCH_2_), 3.92 (s, 3H, NCH_3_), 1.90 – 1.77 (m, 2H, NCH_2_C*H_2_*), 1.39 – 1.32 (m, 2H, NCH_2_CH_2_C*H_2_*), 0.95 (t, J = 7.4 Hz, 3H, CH_3_).


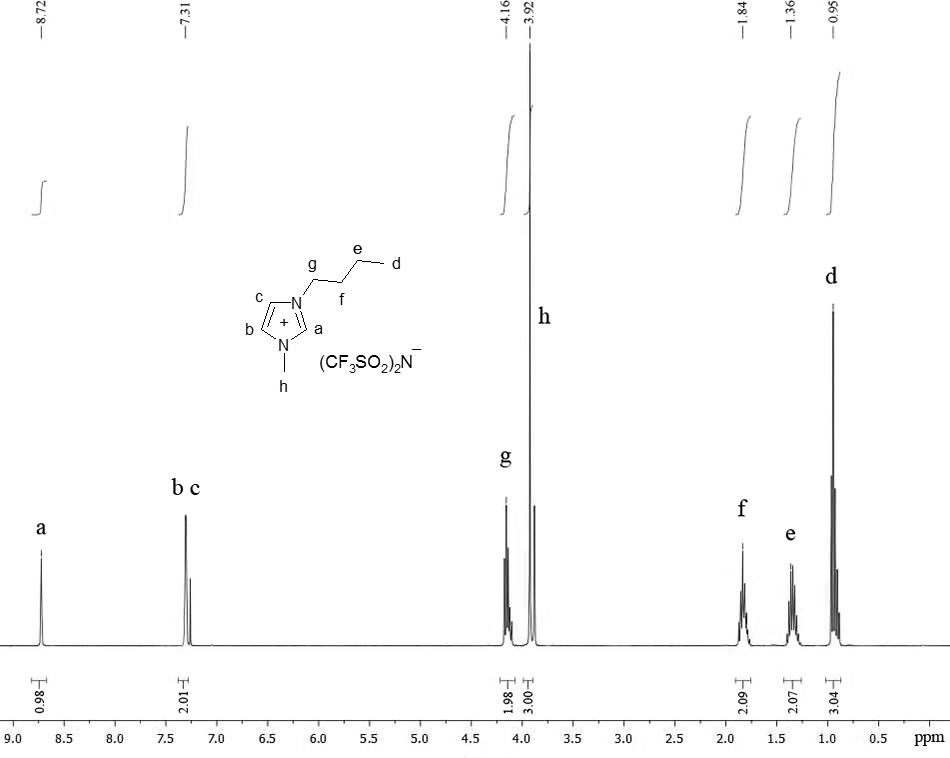


Fig. S3. ^1^H NMR spectrum of [BMIM] [TFSI]
